# Adenoid lymphocyte heterogeneity in pediatric adenoid hypertrophy and obstructive sleep apnea

**DOI:** 10.3389/fimmu.2023.1186258

**Published:** 2023-05-22

**Authors:** Yaxin Zhu, Shengming Wang, Yingchao Yang, Bojun Shen, Anzhao Wang, Xiaoman Zhang, Xiaoxu Zhang, Niannian Li, Zhenfei Gao, Yuenan Liu, Jingyu Zhu, Zhicheng Wei, Jian Guan, Kaiming Su, Feng Liu, Meizhen Gu, Shankai Yin

**Affiliations:** ^1^ Department of Otolaryngology Head and Neck Surgery and Center of Sleep Medicine, Shanghai Sixth People’s Hospital Affiliated to Shanghai Jiao Tong University School of Medicine, Shanghai, China; ^2^ Department of Otolaryngology Head and Neck Surgery, Shanghai Children’s Hospital, Shanghai Jiao Tong University, Shanghai, China

**Keywords:** adenoid hypertrophy, obstructive sleep apnea, T cells, B cells, multicolor flow cytometry

## Abstract

**Introduction:**

Adenoid hypertrophy is the main cause of obstructive sleep apnea in children. Previous studies have suggested that pathogenic infections and local immune system disorders in the adenoids are associated with adenoid hypertrophy. The abnormalities in the number and function of various lymphocyte subsets in the adenoids may play a role in this association. However, changes in the proportion of lymphocyte subsets in hypertrophic adenoids remain unclear.

**Methods:**

To identify patterns of lymphocyte subsets in hypertrophic adenoids, we used multicolor flow cytometry to analyze the lymphocyte subset composition in two groups of children: the mild to moderate hypertrophy group (n = 10) and the severe hypertrophy group (n = 5).

**Results:**

A significant increase in naïve lymphocytes and a decrease in effector lymphocytes were found in severe hypertrophic adenoids.

**Discussion:**

This finding suggests that abnormal lymphocyte differentiation or migration may contribute to the development of adenoid hypertrophy. Our study provides valuable insights and clues into the immunological mechanism underlying adenoid hypertrophy.

## 1 Introduction

Obstructive sleep apnea (OSA) is a common disorder characterized by a recurrent partial or complete collapse of the upper airway during sleep, which leads to hypoxia and sleep fragmentation (1). The prevalence of OSA in children is 1.2% to 5.7% and adenoid hypertrophy is the major cause (2, 3). Without appropriate diagnosis and treatment, it will cause a series of complications, including the adenoid face, cognitive deficits, behavioral abnormalities and growth retardation (3). Adenoidectomy is the standard clinical treatment for moderate to severe adenoid hypertrophy. However, it carries several risks, such as pain, bleeding and anesthetic complications. It is also associated with a significantly higher chance of developing respiratory, allergic, and infectious diseases in adulthood (4). Mild adenoid hypertrophy is usually treated with nasal glucocorticoid or leukotriene receptor antagonists, but the efficacy is limited (5–7). In general, there is still a lack of safe and effective treatments for adenoid hypertrophy in children. Therefore, it is necessary to investigate the immunological mechanism of adenoid hypertrophy.

Adenoid is mucosa-associated lymphoid tissue located in the posterior wall of the nasopharyngeal apex, which is part of Waldeyer’s ring. It contains a large number of lymphoid follicles and diffuse immune cells that form the first line of defense of the immune barrier in the digestive and respiratory tracts (8). Antigens are transported into the adenoid by microfold cells interspersed among epithelial cells. Then antigen presenting cells present exogenous antigens to naïve T cells (TN) located in the interfollicular region of adenoids, which thereafter differentiate into diverse effector T cells and perform immune functions by direct contact or secretion of various cytokines. While naïve B cells (BN) differentiate into plasma cells (PC) after antigens stimulation and T cells help, which mediate humoral immunity through the production and secretion of antibodies (9, 10).

Previous studies have shown that adenoid hypertrophy is related to pathogenic infection and adenoid local immunity disorder, and ultimately manifests as abnormalities in the number and function of various lymphocyte subsets in the adenoid (11–24). However, most studies on adenoid hypertrophy have focused on the changes in the proportion of classic lymphocyte subsets or cytokine levels. The heterogeneity of lymphocyte subsets in hypertrophic adenoids remains poorly characterized (25, 26).

To investigate the lymphocyte heterogeneity of hypertrophic adenoids in children with OSA, we compared the lymphocyte subset composition of adenoids in children with different degrees of hypertrophy using multicolor flow cytometry. A significant increase in naïve lymphocytes and a decrease in effector lymphocytes in severe hypertrophic adenoids in children were observed. Our study provides clues for future studies on the immunological mechanisms of adenoid hypertrophy.

## 2 Materials and methods

### 2.1 Patients and samples

Adenoids were obtained from children who underwent adenoidectomy at the Sixth People’s Hospital of Shanghai Jiao Tong University School of Medicine from September 2022 to October 2022. All patients were diagnosed with OSA and tonsillar hypertrophy, and had been treated with nasal glucocorticoids and/or leukotriene receptor antagonists before surgery. Adenoid hypertrophy was scored by the nasal endoscopic quadrature method proposed by Franco et al (27). It can be divided into four degrees based on the percentage of nostril area obstructed by the adenoid: degree 1 = 0-25%, degree 2 = 26%-50%, degree 3 = 51%-75%, and degree 4 = 76%-100%. Written or verbal informed consent was obtained from the patient’s parents/guardians. Sample collection was approved by the Ethics Committee of the Sixth People’s Hospital of Shanghai Jiao Tong University School of Medicine (2019-KY-050(K)).

### 2.2 Mononuclear cell isolation and cryopreservation

Adenoids were cut into small pieces and ground through a 70 mm filter using the plunger end of a syringe. Then, the cells were washed into a centrifuge tube with PBS. Samples were centrifuged at 4 °C for 5 min at 400 g and the supernatant was discarded. The pellet was resuspended in 3 mL of PBS. Mononuclear cells were obtained by Ficoll density gradient centrifugation (GE Healthcare) and frozen at -80 °C in cell preservation solution.

### 2.3 Multicolor flow cytometry

Frozen mononuclear cells were recovered, and the cell concentration was adjusted to 10^7^ cells/mL. 50 μL sample, 1 μL dead or live dye (BioLegend) and 1.5 mL PBS were mixed well and incubated for 15 min at room temperature protected from light. Cells were washed with 2 mL Cell Staining Buffer (BioLegend) and resuspended in 100 μL Cell Staining Buffer. Then cells were stained with 5 μL Human TruStain FcX (BioLegend) for 10 min at room temperature protected from light and centrifuged at room temperature for 5 min at 400 g. After aspirating 50 μL supernatant, 50 μL mixture including pretitrated antibody, True-Stain Monocyte Blocker (BioLegend) and Brilliant Stain Buffer Plus (BD Pharmingen) (except single-stained tubes) was added and incubated with cells for 20 min at room temperature protected from light. Cells were washed once, resuspended in 300 μL cell staining buffer and acquired on a flow cytometry instrument (Cytek). We designed a 17-color panel to identify major T cell and B cell subsets: TN, T follicular helper cells (Tfh), regulatory cells (Treg), central memory T cells (Tcm), effector memory T cells (Tem), terminally differentiated effector memory T cells (Temra), BN, resting memory B cells (RM), activated memory B cells (AM), follicular B cells (FO B), germinal center B cells (GC B), PC and plasmablasts. Data were downscaled and clustered using FlowJo’s plug-ins UMAP and FlowSom. A complete list of antibodies and staining plates used can be found in **Supplementary Table 1**.

### 2.4 Flow cytometry analysis

Automated compensation was calculated by FCS Express Flow Cytometry software using single stained compensation beads (BioLegend). FCS3.1 files were imported into FlowJo v.10.8.1 (BD Biosciences) and quality controlled using FlowAI (v2.3.1). Cells were gated manually on good events. After adherent cells and dead cells were removed, T cells were gated as CD3^+^ cells, and B cells were gated as CD19^+^CD20^+^ cells. Sample sizes were standardized for each sample using DownSample (v3.3.1) for T cells and B cells, making the samples comparable with each other. The final number of T cells per sample was 8900 and the number of B cells was 14000. T cells and B cells of all samples were combined separately for subsequent analysis. UMAP (v3.1) (Euclidean distance function, nearest neighbor = 15, minimum distance = 0.5) was used for dimensionality reduction and FlowSom (v3.0.18) was used for automated clustering. T cells were divided into 20 clusters and B cells were divided into 10 clusters. Finally, ClusterExplorer (v1.7.4) was used for visualization. Detailed parameters are shown in **Supplementary Table 2**.

### 2.5 Immunofluorescence

The paraffin sections were dewaxed in xylene and ethanol in turn. After washing with dH2O, the section was placed in a pressure cooker filled with citrate buffer (pH 6.0) for antigen retrieval. After natural cooling, the section was washed 3 times with PBS buffer for 5 min each time. Then, the sections were placed in a 3% hydrogen peroxide solution to block endogenous peroxidase for 20 min at room temperature and protected from light. The sections were blocked with serum and incubated at 37 °C with primary antibody and secondary antibody sequentially. After washing with PBS, the sections were incubated with tyramide signal amplification solution at 37 °C for 30 min and followed by antigen retrieval. The same steps were used for staining with the other two antibodies. Finally, DAPI was used to stain the nucleus.

### 2.6 Ki67 score

Randomly selected 63x fields of mantle zone of the lymphatic follicle. The Ki67 score (number of Ki67 positive cells/total number of cells) was calculated for the average of each one hundred B cells in three views.

### 2.7 Statistics

Statistical analysis and graphing were performed in GraphPad Prism Version 9.4.1. The hypothesis test was assessed using the Mann-Whitney U test. Two-tailed P values < 0.05 were considered significant.

## 3 Results

### 3.1 Flow cytometric data analysis workflow

**Figure 1:**
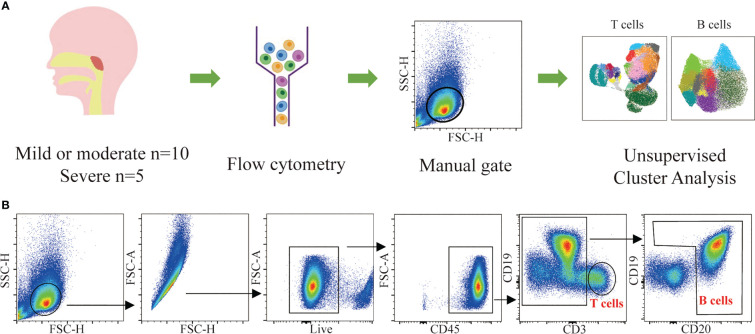
Flow cytometric data analysis workflow. **(A)** Summary of the experimental workflow. **(B)** Gating strategy used for the identification of T cells and B cells and downstream analysis.

To compare the lymphocyte subset composition of adenoids in children with different degrees of hypertrophy, 15 children with OSA and adenoid hypertrophy were included in our study. They were divided into two groups: mild/moderate group with 1 to 3 degree adenoid hypertrophy (n = 10) and severe group with 4 degree adenoid hypertrophy (n = 5). There was no significant difference in age or BMI between the two groups. The baseline characteristics of the patients are shown in **Table 1**. Adenoids were prepared as a single-cell suspension of monocytes for flow cytometry detection. To identify major T cells and B cells subsets in adenoids, a 17-color flow panel (CD45, CD45RA, CD3, CD4, CD8, CD185, CD25, CD19, CD20, IgM, CD27, CD21, CD38, CD197, CD278, CD279, live/dead) was introduced in this study (11). After quality control, T cells and B cells were manually gated (**Figure 1B**). Then T cells and B cells were respectively downscaled, autoclustered and visualized (**Figure 1A**).

**Table 1:** Baseline characteristics of patients.

	Mild to moderate n = 10	Severe n = 5	p
Age (years)	8 (1.91)^a^	7 (2.59)	ns
Height (m)	1.38 (0.11)^a^	1.30 (0.19)	ns
Weight (kg)	35.50 (12.01)^a^	21.00 (17.64)	ns
BMI (kg/m^2^)	16.83 (4.77)^a^	15.29 (5.55)	ns
Nasal glucocorticoids N (%)	10 (100%)	5 (100%)	ns
Leukotriene receptor antagonists N (%)	0 (0%)	2 (40%)	ns
Adenoid size^b^	2.5 (0.53)	4.0 (0)	< 0.001

a Two missing values in this group.

b Adenoid size scored according to the percentage of nostril area obstructed by the adenoid: 1 = 0-25%, 2 = 26%-50%, 3 = 51%-75% and 4 = 76%-100%.

Data are shown as the mean (SD) or the number (percentage). P value calculated using the Mann-Whitney U test and Chi-square test.

ns, no significance.

### 3.2 Heterogeneity of T cells in adenoids of pediatric patients with OSA

**Figure 2:**
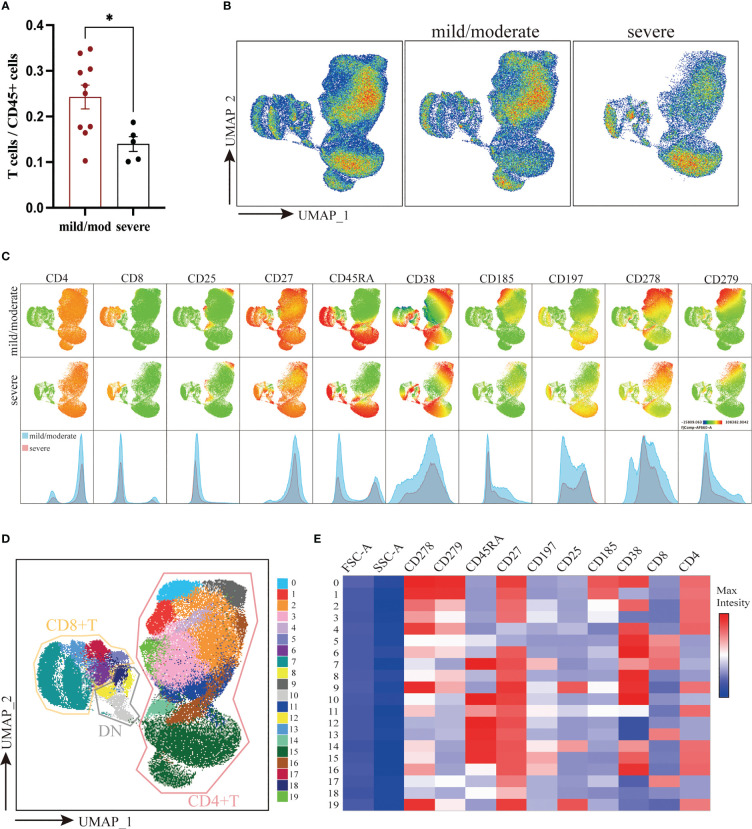
Downscaling and clustering of T Cells in Adenoids of Pediatric Patients with OSA. **(A)** Bar plot of the proportion of T cells to CD45^+^ cells. Bars and error bars indicate the mean ± SEM. Statistical significance was calculated using the Mann-Whitney U test. * p < 0.05. **(B)** UMAP plot of T cells. **(C)** UMAP plots and histograms display the expression of T cell markers in the mild/moderate group and severe group. **(D)** FlowSom clusters overlaid on the UMAP plot. **(E)** Heatmap of marker expression intensity scaled individually FlowSom clusters for each marker.

T cells were defined as live CD45^+^CD3^+^ cells. The proportion of T cells to total CD45^+^ cells was 24.26% ± 2.62% in the mild/moderate group and 14.47% ± 1.61% in the severe group. Compared to the mild/moderate group, the proportion of T cells to total CD45^+^ cells were significantly reduced in the severe group (p < 0.0280) (**Figure 2A**).

To visualize high-parameter datasets in a two-dimensional space, UMAP was performed on 133,500 T cells according to markers of T cells (**Figures 2B, C**) (28). To identify the composition of T cells, 20 clusters were identified using FlowSom and projected on the UMAP plot for visualization (**Figure 2D**). The features of each cluster were revealed by a heatmap (**Figure 2E**). In addition, we calculated the coefficient of variation for each cluster of T cells in both groups to identify the homogeneity of the data **(** **Supplementary Figure 1A** **)**.

According to the expression of CD4 and CD8, 20 identified clusters were split into 3 groups: CD4^+^ T cells (Cluster 0, 1, 2, 3, 4, 9, 11, 14, 15, 16, and 19), CD8^+^ T cells (Cluster 5, 6, 7, 13, and 17) and double negative T cells (DN T) (Cluster 8, 10, 12, and 18) (**Figures 2D, E**). Notably, DN T cells were positioned closer to CD8^+^ T cells on the UMAP plot, indicating that their marker expression was much more similar. Overall, there was no significant difference between the two groups in the ratio of CD4^+^, CD8^+^ and DN T cells (**Supplementary Figure 1B**).

**Figure 3:**
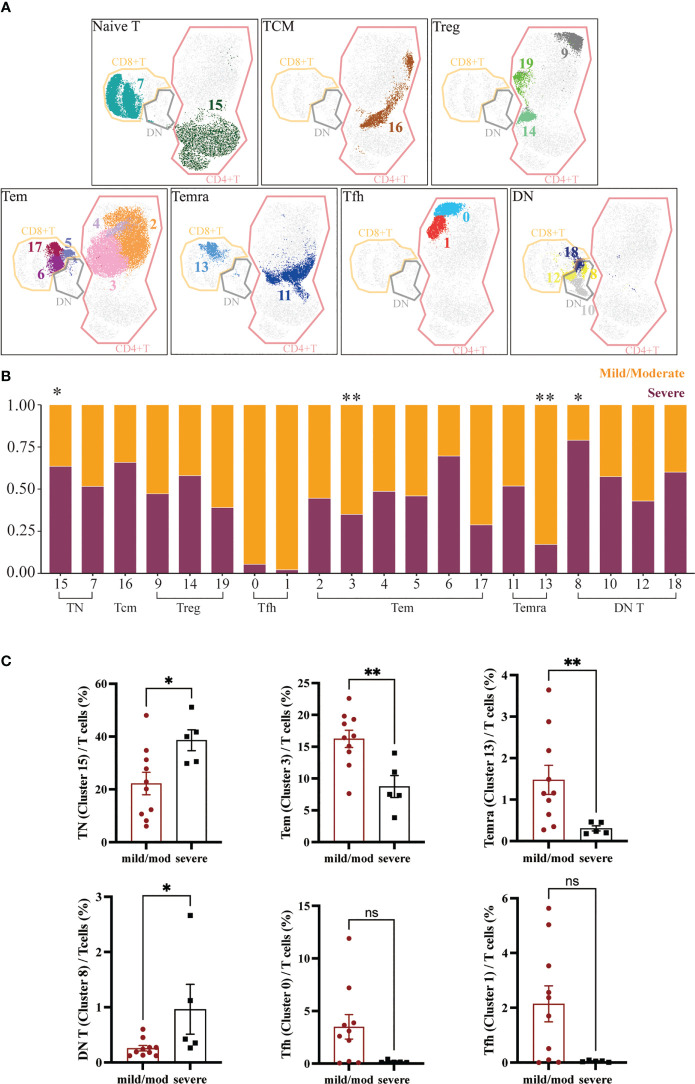
Heterogeneity of T Cells in Adenoids of Pediatric Patients with OSA. **(A)** FlowSom clusters overlaid on the corresponding UMAP separated by functional group. **(B)** Relative abundance of two groups T cells within each FlowSom cluster. *p < 0.05, **p < 0.01. **(C)** Frequency of T cells clusters among T cells. Bars and error bars indicate the mean ± SEM. Statistical significance was calculated using the Mann-Whitney U test.

CD4^+^ T cells were further classified into 6 subgroups according to their different marker expression: CD45RA^+^CCR7^+^CD27^+^CD4^+^ TN (Cluster 15), CD45RA^-^CCR7^+^CD27^+^CD4^+^ Tcm (Cluster 16), CD25^+^ Treg (Cluster 9, 14, 19), CXCR5^+^ Tfh (Cluster 0, 1), CD45RA^-^CCR7^-^CD27^+/-^CD4^+^ Tem (Cluster 2, 3, 4) and CD45RA^+^CCR7^-^CD27^-^CD4^+^ Temra (Cluster 11). Between the mild/moderate groups and severe groups, the frequencies of Tcm, Tfh, Treg, and Temra clusters were not significantly difference. However, the proportion of PD1^lo^CD38^lo^ Tem (Cluster 3) to T cells in mild/moderate group and severe group was 16.55% ± 1.35% and 7.52% ± 1.73% respectively. Compared to the mild/moderate group, it was significantly reduced in the severe group (p = 0.0047). The proportion of TN (Cluster 15) to T cells was 21.55% ± 4.25% in the mild/moderate group and 40.00% ± 3.94% in the severe group, which was significantly elevated in the severe group (p = 0.0400) (**Figures 3A-C**).

Similarly, CD8+ T cells were also classified into 3 subgroups according to marker expression: CD45RA^+^CCR7^+^CD27^+^CD8^+^ TN (Cluster 7), CD45RA^-^CCR7^-^CD27^+/-^CD8^+^ Tem (Cluster 5, 6, 17) and CD45RA^+^CCR7^-^CD27^-^CD8^+^ Temra (Cluster 13). The proportion of CD8^+^ T cells clusters to T cells did not differ significantly between groups except CD8^+^ Temra (Cluster 13), which was significantly lower in the severe group (p = 0.0080), 1.16% ± 0.35% in the mild/moderate group and 0.24% ± 0.06% in the severe group (**Figures 3A-C**).

DN T cells were divided into four clusters (Cluster 8, 10, 12, 18). The proportion of CD45RA^-^CD38^+^ DN T cells (Cluster 8) to DN T cells was significantly increased in the severe group (p = 0.0373), which was 0.22% ± 0.05% in the mild/moderate group and 0.42% ± 0.45% in the severe group (**Figures 3A-C**).

### 3.3 Heterogeneity of B cells in adenoids of pediatric patients with OSA

**Figure 4:**
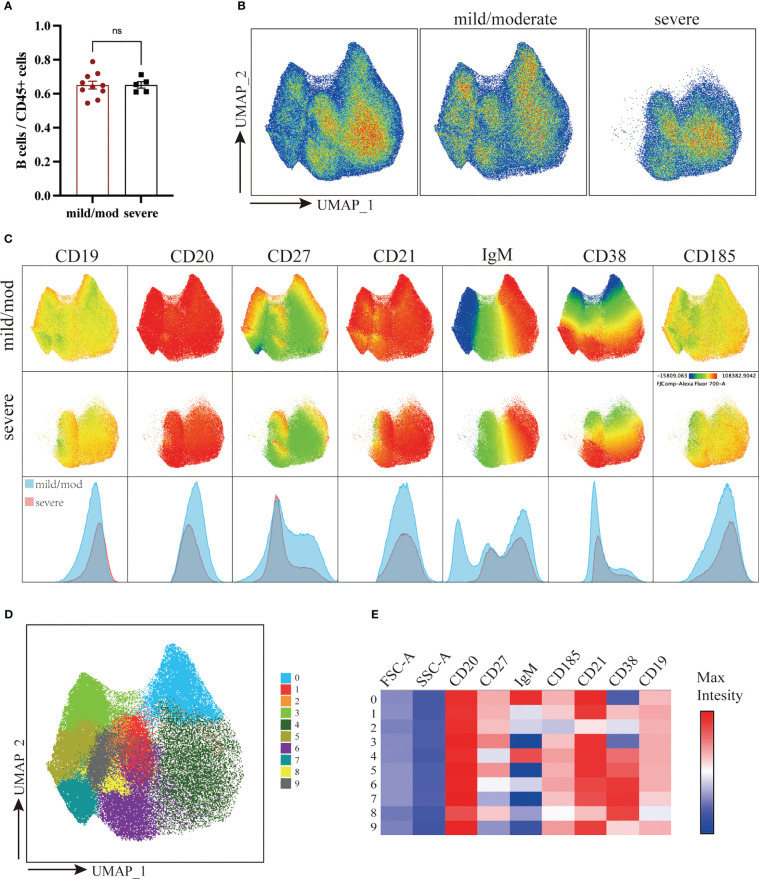
Downscaling and clustering of B Cells in Adenoids of Pediatric Patients with OSA. **(A)** Bar plot of the proportion of B cells to CD45^+^ cells. Bars and error bars indicate the mean ± SEM. Statistical significance was calculated using the Mann-Whitney U test. ^ns^ p > 0.05. **(B)** UMAP plot of B cells. **(C)** UMAP plots and histograms display the expression of B cell markers in the mild/moderate group and severe group. **(D)** FlowSom clusters overlaid on the UMAP plot. **(E)** Heatmap of marker expression intensity scaled individually FlowSom clusters for each marker.

B cells were defined as live CD45^+^CD3^-^CD19^+^ or CD45^+^CD3^-^CD20^+^ cells. The proportion of B cells to total CD45^+^ cells was 64.47% ± 2.30% in the mild/moderate group and 65.35% ± 1.91% in the severe group. There was no significant difference between the two groups (**Figure 4A**). A total of 210,000 B cells were analyzed using the same method according to the marker expression of B cells (**Figures 4B-E**) (29). B cells were divided into 10 clusters and the coefficient of variation for each cluster was calculated (**Supplementary Figure 1C**).

**Figure 5:**
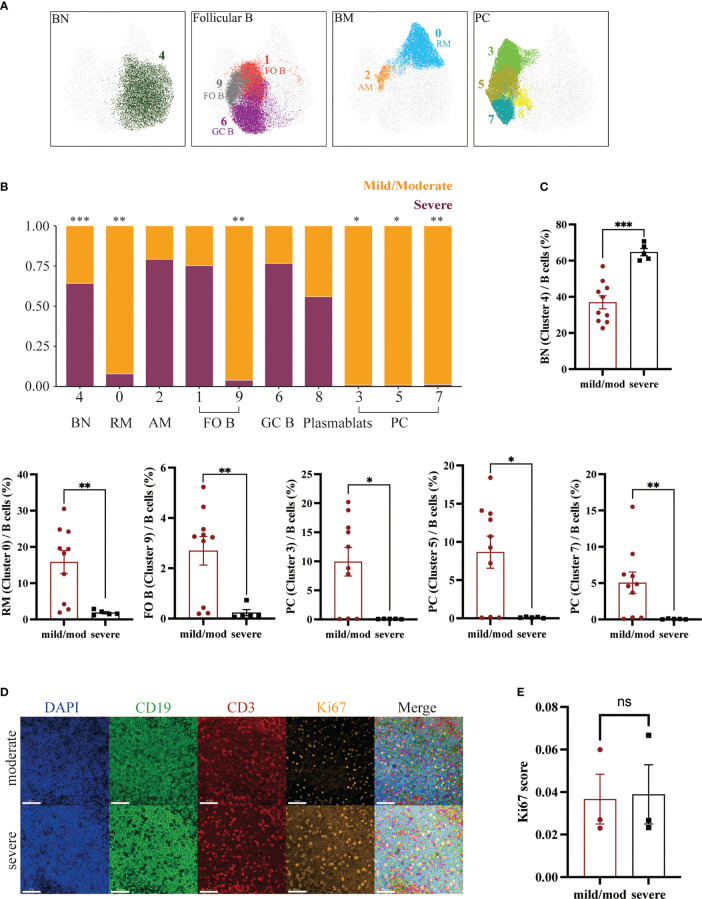
Heterogeneity of B Cells in Adenoids of Pediatric Patients with OSA. **(A)** FlowSom clusters overlaid on the corresponding UMAP separated by functional group. **(B)** Relative abundance of two groups of B cells within each FlowSom cluster. *p < 0.05, **p < 0.01, ***p < 0.001. **(C)** Frequency of BN among B cells. Bars and error bars indicate the mean ± SEM. Statistical significance was calculated using the Mann-Whitney U test. **(D)** Immunofluorescence images of moderate hypertrophy and severe hypertrophy adenoids. DAPI, blue; CD19, green; CD3, red; Ki67, orange. 63x fields. Scale bars: 50 μm. **(E)** Bar plot of the Ki67 score. Bars and error bars indicate the mean ± SEM. Statistical significance was calculated using the Mann-Whitney U test.

They can be divided into 7 groups: IgM^+^CD27^-^CD21^+^CD38^+^ BN (Cluster 4), CD27^+^CD21^+^CD38^-^ RM (Cluster 0), CD27^+^CD21^lo^CD38^-^ AM (Cluster 2), CD21^+^CD185^+^CD38^lo^ FO B (Cluster 1, 9), CD27^+^CXCR5^+^CD38^hi^ GC B (Cluster 6), IgM^-^CD38^+^ PC (Cluster 3, 5, 7) and IgM^lo^CD38^+^ plasmablast (Cluster 8) (**Figure 5A**). The proportion of BN (Cluster 4) to B cells in the mild/moderate group and severe group was 35.55% ± 3.59% and 63.80% ± 2.01% respectively, and it was significantly increased in the severe group (p = 0.0007). While RM (cluster 0), FO B (cluster 9) and PC (cluster 3, 5, 7) were significantly decreased in the severe group (p = 0.0047, 0.0047, 0.0193, 0.0263 and 0.0013, respectively) (**Figures 5B, C**; **Supplementary Figure 1D**).

In addition, we performed immunofluorescence on three mild or moderate and three severe hypertrophic adenoids (**Figure 5D**). The Ki67 index of B lymphocytes in the mantle zone (mainly BN) was also calculated and showed no significant difference (**Figure 5E**) (30).

## 4 Discussion

To investigate the lymphocyte heterogeneity of hypertrophic adenoids in children with OSA, we compared the lymphocyte subset composition of adenoids in children with different degrees of hypertrophy using multicolor flow cytometry. Two groups of children with adenoid hypertrophy were included: a mild/moderate hypertrophy group and a severe hypertrophy group. Lymphocytes from their adenoids were detected using a 17-color flow panel. This panel was able to identify the major subsets of T cells and B cells. Then, the data were analyzed with an automated clustering method. The results suggested that the proportion of T cells to lymphocytes significantly decreased and the proportion of B cells increased as the degree of adenoid hypertrophy increased. This prompted us to further explore whether there were differences in the subsets of T cells and B cells.

T cells in the adenoids of children are mainly composed of three types: CD4^+^ T cells, CD8^+^ T cells and DN T cells. Several subsets of CD4^+^ T cells were identified including CD4^+^ TN, CD4^+^ Tcm, Treg, Tfh, CD4^+^ Tem and CD4^+^ Temra. In comparing clusters of T cells between the two groups, we found a significant reduction in PD1^lo^CD38^lo^CD4^+^ Tem in the severe group. Low expression of PD1 and CD38 suggested that this was a more resting-state population of Tem. Studies have shown that CD4^+^ Tem is derived from Th1, Th2 and Th17, which can produce multiple cytokines within hours after TCR stimulation (31, 32). In addition, a significant increase in CD4^+^ TN was observed. According to the results, we speculated that the decrease in CD4^+^ Tem may be due to decreased TN differentiation from Th to Tem. In CD8^+^ T cells, we also identified several continuously differentiated subsets, which were CD8^+^ TN, CD8^+^ Tem and CD8^+^ Temra, respectively. There was a significant decrease in CD8^+^ Temra in the severe group. Temra is Tem expressing CD45RA, a terminally differentiated Tem (33). Persistent viral infection, inflammation and aging will induce the accumulation of Temra (34). Although the proliferative capacity of Temra is limited, it can be activated and proliferate if certain co-stimulatory signals are provided and rapidly die after performing effector functions (35, 36). In addition, we found a cluster of DN T cells was increased in the severe group. DN T cells represent a separate lineage with distinct phenotypes and unique properties (37). DN T cells in the periphery are unable to proliferate and mainly converted from CD8^+^ T cells. They can re-express CD8 when lymphocytes are reduced (38). This is consistent with our observation in the UMAP plot that DN T cells were closer to CD8^+^ T cells. Most DN T cells display a phenotype of terminally differentiated effector cells capable of producing cytokines such as IFN-γ and IL-10 (39). Previous studies have shown that increased DN T cells can lead to lymphocyte proliferation and inflammation and cytokines such as IFN-γ and IL-10 were elevated in hypertrophic adenoids of children (40, 41). Based on our results we hypothesized that there was increased differentiation of CD8^+^ T cells to DN T cells in hypertrophic adenoids, which eventually led to a decrease in effector CD8^+^ T cells and an increase in pro-inflammatory DN T cells and proliferation of lymphocytes. In conclusion, we found abnormal differentiation of T cells which may cause immune disorder in hypertrophic adenoids of children.

In addition, we identified several constantly differentiating B cells subsets: BN, RM, AM, FO B, GC B, PC and plasmablasts. BN will differentiate into FO B and GC B as it migrates toward the germinal center. GC B can develop into plasmablasts and finally into PC to exert immune efficacy by producing antibodies. GC B can differentiate into BM as well. After being stimulated by a particular antigen, RM can rapidly activate to become AM and exert immune function (9). Among B cells clusters, BN was significantly increased in the severe group. In contrast, FO B, RM and PC, which exert effector functions, were significantly reduced. This is similar to the results of a study in tonsillar hypertrophy. Carrasco et al. found an increase in BN in children with large tonsils and a discrepancy with the expression of Ki67, which represents the level of cell proliferation, suggesting that the increase in BN may be due to the accumulation of cells caused by impaired differentiation (11). Similarly, our immunofluorescence results showed no difference in Ki67 expression between the two groups, suggesting that the increase in BN may be the result of impaired differentiation or migration instead of proliferation.

Similar to a previous study, we did not find a correlation between Tfh and the degree of adenoid hypertrophy (12). Moreover, despite the lack of statistical significance in the comparison of the Tfh proportion between the two groups, we observed a very low frequency of Tfh in the severe group. Likewise, plasma cells were also scarce in the severe group. Tfh is essential for the formation and maintenance of germinal centers where rapidly proliferating B cells undergo somatic mutation and the selection and eventual differentiation into BM or long-lived PC (42). Hence, the reduced proportions of both Tfh and PC could be attributed to the following factors: 1) The decline in Tfh cell numbers due to various causes resulted in the diminished generation of plasma cells. 2) The expansion of other lymphocyte subsets led to a relative decrease. 3) BN differentiation disorder. Exploration of the specific mechanism is beyond the scope of this study and warrants further investigation in the future.

Previous studies on adenoid hypertrophy have mostly focused on classical lymphocytes or cytokines. Our study is the first to use multicolor flow cytometry to compare the lymphocyte subset composition of adenoids in children with different degrees of hypertrophy. We analyzed the data with an automated clustering method, which avoids the limitations of manual circle gates and provides a more comprehensive understanding of the composition of lymphocytes in hypertrophic adenoids. Our results suggest an accumulation of naïve lymphocytes and a decrease in effector cells in the hypertrophic adenoids. This may be due to impaired differentiation of T cells and B cells.

Finally, there are still many shortcomings in our study. First, the sample size included in our study was small. The coefficient of variation of clusters with fewer cells was large. And the range of the ages of patients was narrow. Future validation of a large sample with a wider range of ages could be performed based on the results. Second, all patients in this study have been treated with nasal glucocorticoids or leukotriene receptor antagonists before surgery which could have different impact on the lymphocyte subsets composition. Nasal glucocorticoids have strong anti-inflammatory and immunosuppressive effects which can lead to the suppression of inflammatory cytokines and the apoptosis of lymphocytes (43). While leukotriene receptor antagonists are non-steroidal anti-inflammatory drugs. The cysteinyl leukotrienes are highly potent mediators of inflammation, resulting in microvascular permeability and inflammatory cell chemotaxis (particularly eosinophils) (44). More detailed groupings can be made according to the patient’s treatment. Third, no functional or transcriptional level studies have been performed. Lymphocyte differentiation is regulated by a very complex transcriptional regulation (45). Further research can be performed at the transcriptional level in the future. Fourth, many studies have shown that the function of lymphocytes is affected by bacteria or viruses (21). For example, human rhinovirus can enter B lymphocytes and form viral replication centers and induce the proliferation of B cells (46, 47). The interaction between lymphocytes and pathogens can be further explored.

## 5 Conclusion

In conclusion, our study explored adenoid lymphocyte heterogeneity in pediatric OSA patients with different degrees of hypertrophy using multicolor flow cytometry. The results revealed an increase in naïve lymphocytes and a decrease in effector lymphocytes. This study provides a clue for future studies on the immunological mechanisms of adenoid hypertrophy.

## Data availability statement

The raw data supporting the conclusions of this article will be made available by the authors, without undue reservation.

## Ethics statement

The studies involving human participants were reviewed and approved by the Ethics Committee of the Sixth People’s Hospital of Shanghai Jiao Tong University School of Medicine (2019-KY-050(K)). Written informed consent to participate in this study was provided by the participants’ legal guardian/next of kin.

## Author contributions

YZ performed experiments, analyzed data, and drafted the manuscript. SW selected the patient cohort. YY collected clinical information. BS collected clinical information. AW contributed to data analysis and data interpretation. XMZ collected clinical information. XXZ contributed to the experiments. NL contributed to data analysis. ZG contributed to data interpretation. YL performed experiments. JZ contributed to data interpretation. ZW contributed to the experiments. JG formulated research questions. KS concepted and designed the work. FL concepted and designed the work, interpreted the data, and revised the manuscript. MG concepted and designed the work. SY concepted and designed the work, interpreted the data, and revised the manuscript. All authors contributed to the article and approved the submitted version.
