# Supplementary material for: Adenoid lymphocyte heterogeneity in pediatric adenoid hypertrophy and obstructive sleep apnea
Source: Front Immunol. 2023 May 22;14:1186258. doi: 10.3389/fimmu.2023.1186258 (PMC10239814; doi:10.3389/fimmu.2023.1186258)
Supplement: Supplementary file 1 [file DataSheet_1.docx]

Supplementary Material

Adenoid Lymphocyte Heterogeneity in Pediatric Adenoid Hyperplasia and Obstructive Sleep Apnea

**Yaxin Zhu^†^, Shengming Wang^†^, Yingchao Yang, Bojun Shen, Anzhao Wang, Xiaoman Zhang, Xiaoxu Zhang, Niannian Li, Zhenfei Gao, Yuenan Liu, Jingyu Zhu, Zhicheng Wei, Jian Guan, Kaiming Su, Feng Liu, Meizhen Gu^‡*^, Shankai Yin^‡*^**

*** Correspondence:**

Meizhen Gu: [gumz@shchildren.com.cn](mailto:gumz@shchildren.com.cn)

Shankai Yin: [skyin@sjtu.edu.cn](mailto:skyin@sjtu.edu.cn)

^†^These authors contributed equally to this work and share first authorship

^‡^These authors contributed equally to this work and share last authorship

# Supplementary Figures and Tables

## Supplementary Figure


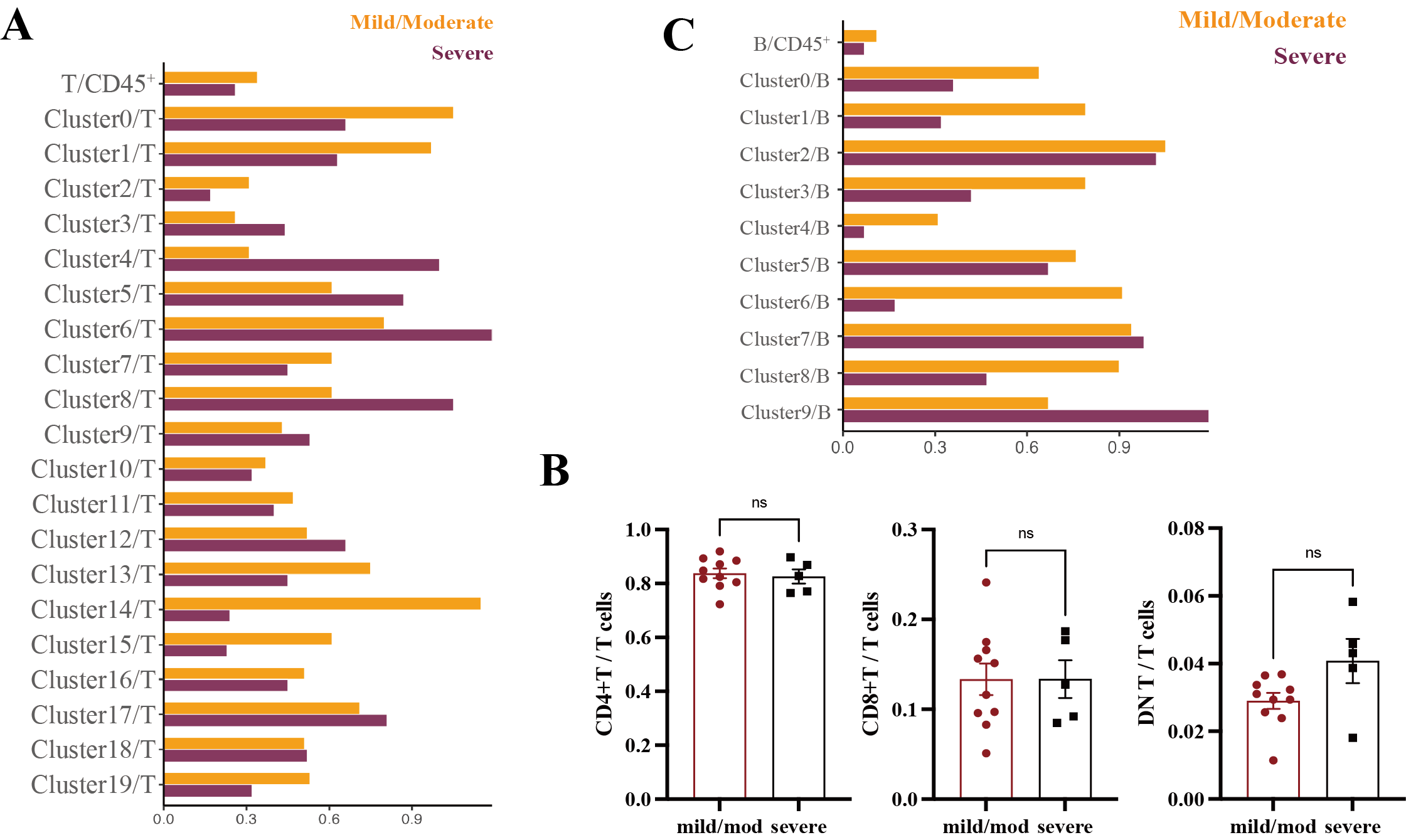


**Supplementary Figure 1.** **(A)** The coefficient of variation for each cluster of T cells in mild/moderate and severe group. **(B)** Bar plots of proportion of CD4^+^ T, CD8^+^ T and DN T cells to total T cells. Bars and error bars indicate the mean ± SEM. Statistical significance was calculated using the Mann-Whitney U test. ^ns^ p > 0.05. **(C)** The coefficient of variation for each cluster of B cells in mild/moderate group and severe group.


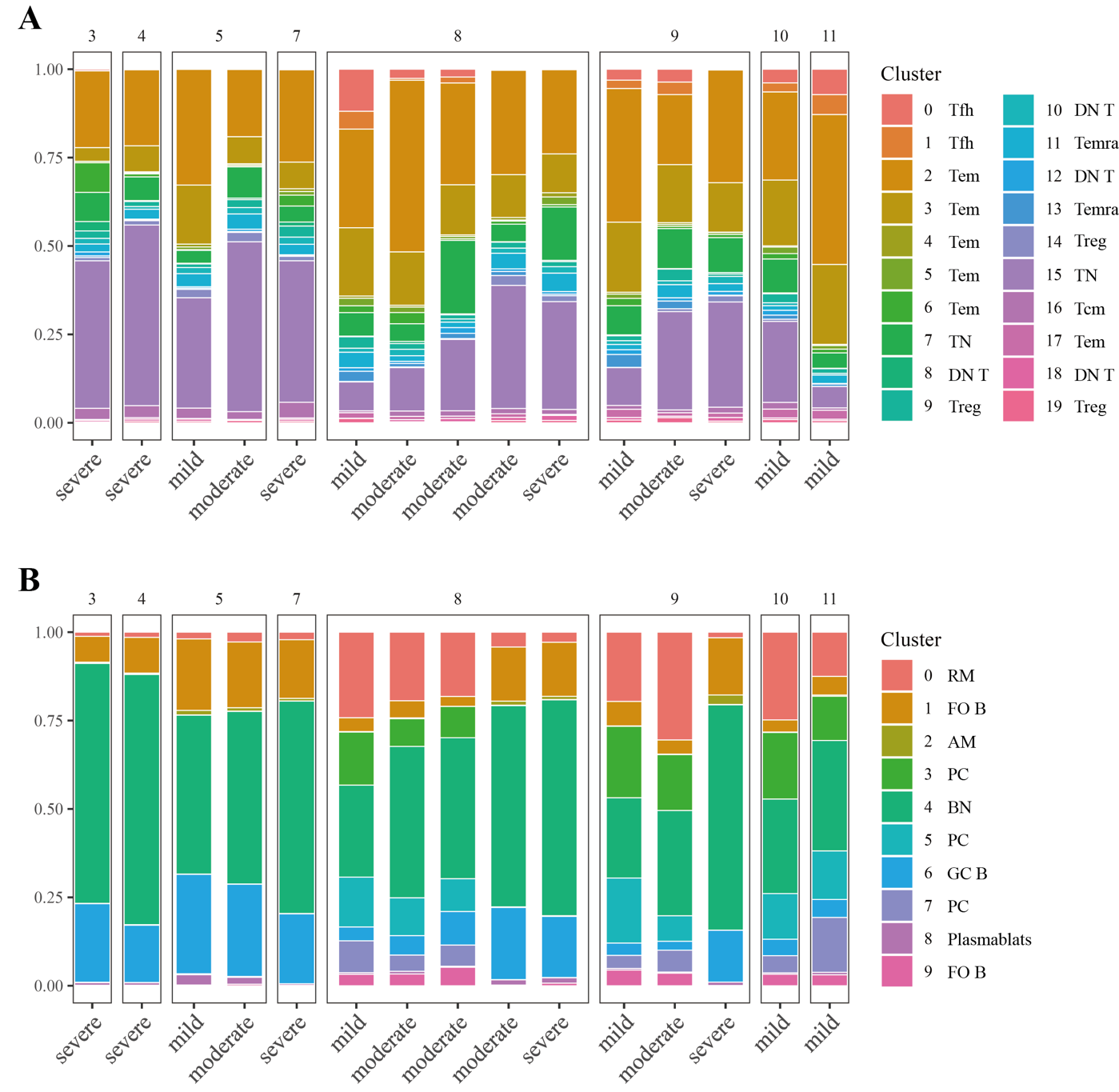


**Supplementary Figure 2.** **(A)** Relative abundance of FlowSom clusters of T cells within each sample ordered by age. **(B)** Relative abundance of FlowSom clusters of B cells within each sample ordered by age.

## Supplementary Tables

| Manufacturer | Cat# | Antibodies | Clone | Channel |
| --- | --- | --- | --- | --- |
| Biolegend | 302281 | Spark Violet™ 423 anti-human CD19 | HIB19 | V2 |
| Biolegend | 353259 | KIRAVIA Blue 520™ anti-human CD197 | G043H7 | B2 |
| Biolegend | 313509 | APC anti-human/mouse/rat CD278 (ICOS) | C398.4A | R1 |
| Biolegend | 344673 | Spark Violet™ 538 anti-human CD4 | SK3 | V8 |
| Biolegend | 314543 | Brilliant Violet 785™ anti-human IgM | MHM-88 | V15 |
| Biolegend | 367421 | Brilliant Violet 421™ anti-human CD279 | NAT105 | V1 |
| Biolegend | 302605 | PE anti-human CD25 | BC96 | B4 |
| Biolegend | 344709 | PerCP/Cyanine5.5 anti-human CD8 | SK1 | B9 |
| Biolegend | 344855 | Alexa Fluor® 660 anti-human CD3 | SK7 | R3 |
| Biolegend | 302833 | Brilliant Violet 711™ anti-human CD27 | O323 | V13 |
| Biolegend | 356951 | PE/Cyanine5 anti-human CD185 (CXCR5) | J252D4 | B8 |
| Biolegend | 302322 | Alexa Fluor® 700 anti-human CD20 | 2H7 | R4 |
| Biolegend | 397225 | PE/Fire™ 810 anti-human CD38 | S17015F | B14 |
| Biolegend | 304061 | APC/Fire™ 750 anti-human CD45 | HI30 | R7 |
| Biolegend | 304133 | Brilliant Violet 605™ anti-human CD45RA | HI100 | V10 |
| Biolegend | 354911 | PE/Cyanine7 anti-human CD21 | Bu32 | B13 |
| Biolegend | 423101 | Zombie Aqua™ Fixable Viability Kit | - | V7 |

**Supplementary Table 1.** Antibodies used in this study.

| T cells | | B cells | |
| --- | --- | --- | --- |
| UMAP | FlowSom | UMAP | FlowSom |
| CD197 | CD197 | IgM | IgM |
| CD278 | CD278 | CD27 | CD27 |
| CD279 | CD279 | CD38 | CD38 |
| CD25 | CD25 | CD21 | CD21 |
| CD27 | CD27 | CD185 | CD185 |
| CD185 | CD185 | CD20 | CD20 |
| CD38 | CD38 | CD19 | CD19 |
| CD45RA | CD45RA |  |  |
| CD4 | CD4 |  |  |
| CD8 | CD8 |  |  |

**Supplementary Table 2.** Markers used in UMAP and FlowSom.
